# Effects of Kelulut Honey on Oestrus Cycle Regulation and Histomorphological Changes in Letrozole-Induced Polycystic Ovary Syndrome Rats: A Preliminary Study

**DOI:** 10.3390/life12060890

**Published:** 2022-06-14

**Authors:** Datu Agasi Mohd Kamal, Siti Fatimah Ibrahim, Azizah Ugusman, Mohd Helmy Mokhtar

**Affiliations:** 1 Department of Physiology, Faculty of Medicine, Universiti Kebangsaan Malaysia, Kuala Lumpur 56000, Malaysia; agasi.mk@ums.edu.my (D.A.M.K.); timi@ukm.edu.my (S.F.I.); dr.azizah@ppukm.ukm.edu.my (A.U.); 2 Department of Biomedical Sciences, Faculty of Medicine and Health Sciences, University Malaysia Sabah, Kota Kinabalu 88400, Malaysia

**Keywords:** PCOS, Kelulut honey, ovary, oestrous cycle

## Abstract

Polycystic ovary syndrome (PCOS) is a complex reproductive, metabolic, and endocrine disorder that affects women of reproductive age. Kelulut honey is stingless bee honey that possesses anti-inflammatory, anti-cancer, anti-diabetic, and potent antioxidative activities in most conditions. However, its value in improving PCOS remains to be elucidated. Thus, this preliminary study aimed to determine the effective dose of Kelulut honey in oestrus cycle regulation and ovarian histomorphological changes in letrozole-induced PCOS rats. PCOS was induced in all-female Sprague Dawley (SD) rats with 1 mg/kg/day of letrozole except for the control group for 21 days. Kelulut honey was then orally administered to the PCOS rats at the dose of 0.5, 1, or 2 g/kg/day, respectively, for 35 days. The oestrous cycle was determined through vaginal smears, while ovarian histomorphological changes were observed by haematoxylin and eosin (H&E) staining. The untreated PCOS rats were characterised by irregular oestrous cyclicity, hyperglycaemia, and aberrant ovarian histology. In this study, Kelulut honey (1 g/kg/day) increased the number of corpus luteum and antral follicles (*p* < 0.05), improved the cystic follicle, and normalised the oestrus cycle (*p* < 0.05). This preliminary study demonstrated that Kelulut honey, particularly at a dose of 1 g/kg/day, has the potential to alleviate oestrus cycle dysregulation and ovarian histomorphological changes occurring in PCOS.

## 1. Introduction

Polycystic ovary syndrome (PCOS) is a complex disorder of reproductive, endocrine, metabolic, and psychological health issues [1]. Its prevalence may reach up to 20% in women of reproductive age, depending on the diagnostic criteria [2]. Women with PCOS may experience hyperandrogenism, irregular menstrual cycles, dyslipidaemia, excessive body weight, hirsutism, and infertility [3]. Infertility has been reported in 40% of women with PCOS [1]. In addition, an estimated 80% of women with anovulatory infertility have PCOS, making PCOS the most common cause of anovulatory infertility [3,4].

Despite its high prevalence and health implications, PCOS is still underdiagnosed, with its underlying aetiology and pathophysiology still not fully clarified [5,6]. The most suggested aetiology of PCOS is abnormalities of ovarian steroidogenesis, which lead to functional ovarian hyperandrogenism [3,7]. Other pathophysiologic abnormalities include insulin-resistant, hyperinsulinism, gonadotrophin secretion abnormalities, and follicular arrest [8]. Hyperandrogenism in PCOS disrupts the physiological rhythm of the normal hormonal cycle in women, hence affecting the menstrual cycle. Hyperandrogenism also affects the ovary in PCOS, manifested mainly by the increased number of subcapsular follicle cysts, follicular arrest, decreased thickness of the granulosa layer, and absence of corpus luteum [9]. Additionally, oxidative stress and low-grade inflammation are also reported to be involved in the pathogenesis of PCOS [10,11]. A systematic review and meta-analysis, which involved 4933 PCOS patients and 3671 controls, found that circulating markers of oxidative stress are impaired in women with PCOS, which suggests that oxidative stress is involved in the pathophysiology of PCOS [12]. Interestingly, the systematic review also found that the levels of oxidative stress markers in women with PCOS are independent of weight excess, indicating the specificity of oxidative stress toward PCOS.

In the meantime, PCOS management in routine clinical practise remains inconsistent [13]. Until now, there has been no definitive cure for PCOS. Thus, PCOS is usually treated based on the symptoms and focuses on disease management [14]. Current drug treatment for PCOS includes clomiphene citrate for ovulation induction and metformin for diabetes management [15]. However, these drugs appear to be associated with several adverse effects, including diarrhoea, vaginal and uterine bleeding, breast tenderness, hot flashes, and abdominal pain [16].

A systematic review by Ismail et al. (2021) recently showed that honey supplementation provides oestrogenic, antioxidative, and anti-inflammatory effects on the female reproductive system [17]. Various types of honey have been investigated for their beneficial effects on the female reproductive system, and one of them is Kelulut honey. Kelulut honey is stingless bee honey, which is reported to have anti-inflammatory, anti-cancer, anti-diabetic, and excellent antioxidative effects in most conditions [18]. Studies around the globe reported that stingless bee honey, including Kelulut honey, contains higher antioxidant content and activity than the regular honey bee, the Apis spp. [19,20]. It has been shown that Kelulut honey contains numerous phenolic and flavonoid compounds, including gallic acid, caffeic acid, syringic acid, catechine, apigenin, chrysin, and quercetin [19,21,22]. Besides that, previous studies have evaluated the composition of Malaysian Kelulut honey, which is demonstrated to be rich in valuable amino acids, polyphenol compounds and good quality of physicochemical and antioxidant properties [19,21,23,24]. Moreover, a recent study showed that disaccharide trehalulose, a highly active antioxidant, is a major component of Kelulut Honey [25]. In addition, Kelulut honey, through its antioxidative effect, was shown to prevent damage to sperm and testis in diabetic rats [26]. However, Kelulut honey has not been explored for its value in improving PCOS. The antioxidative property of Kelulut honey may have the potential to be used as an adjuvant to complement the currently available treatment for PCOS. Thus, this study aimed to determine the effective dose of Kelulut honey in regulating the oestrus cycle and histomorphological changes of the ovary in letrozole-induced PCOS in rats. We hypothesised that Kelulut honey treatment could improve oestrous cycle regularities and alleviate ovarian histomorphological derangement.

## 2. Materials and Methods

### 2.1. Honey Sample

An experienced local beekeeper harvested the Kelulut honey in Negeri Sembilan, Malaysia. The bees gathered nectar from the nearby herbal plant. The honey was maintained unprocessed and stored in amber bottles at room temperature, away from direct sunlight and heat sources.

### 2.2. Animal Preparation

This study was approved by the National University of Malaysia Animal Ethics Committee (Ethical Approval Code FISIO/FP/2020/MOHD HELMY/14-MAY/1104-JUNE-2020-MAY-2023). Female Sprague–Dawley (SD) rats weighing 120–150 g that showed at least two consecutive regular oestrus cycles were used in this study. The rats were obtained from the Laboratory Animal Resources Unit, Faculty of Medicine, National University of Malaysia. All rats were individually caged, acclimatised for one week, maintained under a 12 h light and 12 h dark cycle in an air-conditioned room at 24 ± 2 °C, and given standard food pellets and water ad libitum. Throughout the treatment period, animals were weighed twice a week, and vaginal smear was observed daily under a light microscope to identify the oestrus stage.

### 2.3. Animal Treatment

**Figure A1:**
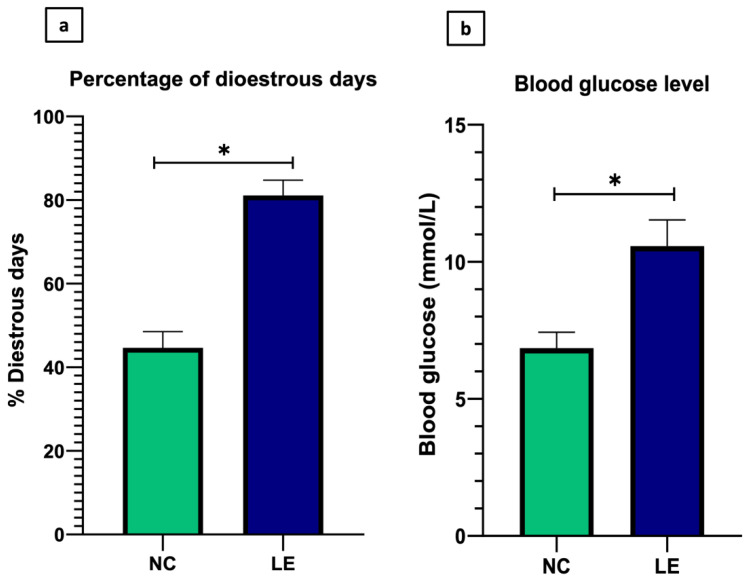
Effects of letrozole treatment on (**a**) percentage of dioestrous days and (**b**) blood glucose levels. NC: normal control; LE: letrozole-induced PCOS. * *p* < 0.05 compared with normal control group, *n* = 4 per group.

**Figure A2:**
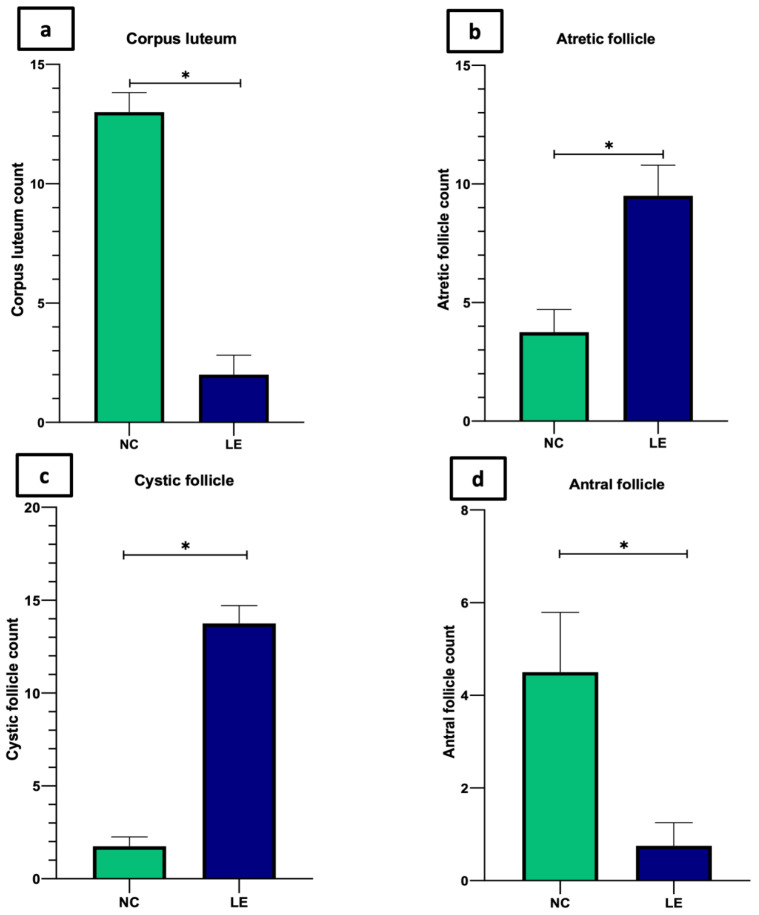
Effects of letrozole treatment on (**a**) corpus luteum, (**b**) atretic follicle, (**c**) cystic follicle, and (**d**) antral follicle. NC: normal control; LE: letrozole-induced PCOS. * *p* < 0.05 compared with normal control group, *n* = 4 per group.

Female rats with the above-mentioned regular oestrus cycles were chosen and divided into two groups. The first group (control group, *n* = 4) received vehicles (distilled water) throughout the study (56 days). The second group (*n* = 20) was given 1 mg/kg/day of letrozole orally, once daily for 21 days, to induce PCOS according to the method described by Kafali et al. [27]. Daily vaginal smears were obtained and examined under Olympus BX40 light microscope (Olympus Corporation, Tokyo, Japan) to determine the oestrus stage. In addition, the rats’ body weight was recorded every week. The rats were confirmed to develop PCOS by oestrus cycle irregularities. Besides, PCOS rats showed the critical characteristics of PCOS, including hyperglycaemia, hyperandrogenism, and numerous cysts, as previously reported [28]. Four PCOS rats (letrozole-induced PCOS group) were sacrificed at the end of the induction period (21 days) to confirm successful PCOS induction. PCOS was successfully induced in all rats treated with 1 mg/kg/day of letrozole for 21 days, as evidenced by the changes in the percentage of dioestrous days, blood glucose levels, and ovarian histomorphological parameters (Figure A1 and Figure A2).

The other PCOS rats (*n* = 16) were then randomly divided into four experimental groups (*n* = 4 per group): Untreated PCOS rats that received distilled water (PCOS), PCOS rats treated with 0.5 g/kg/day of Kelulut honey (KH0.5), PCOS rats treated with 1 g/kg/day of Kelulut honey (KH1), and PCOS rats treated with 2 g/kg/day of Kelulut honey (KH2) orally for 35 days, respectively. Treatment duration and doses of Kelulut honey given were based on the study by Sahlan et al. [29]. At the end of the 35-day period, all the animals were sacrificed by ketamine-xylazine overdose (0.3 mL/100 g body weight) [30]. The ovaries were preserved in 10% buffered formalin and processed for histomorphological analysis.

### 2.4. Determination of Fasting Blood Glucose

This test was carried out prior to sacrificing the rats. Rats were fasted for eight hours, and blood glucose was determined using a handheld glucometer in a tail blood sample (Accucheck performa, Roche Diagnostics, Basel, Switzerland).

### 2.5. Determination of Oestrous Cycle

Vaginal smears were carried out on all the rats at 9:00 am daily using cotton bud immersed in 0.9% saline. A cotton bud was then rolled to the glass slide to collect the vaginal secretion. Oestrous cycles were tracked until the end of the study. Methylene blue was used to stain the cells, and a light microscope was used to examine them. As previously reported, the smears were classified as one of the four oestrous cycle stages [31]. The proestrous phase is a smear with a high percentage of rounded and nucleated epithelial cells. In contrast, the smears with predominantly irregular cornified cells without a nucleus are considered the oestrous phase. Leukocytes were small, round cells with no nucleus, primarily in the dioestrous phase. Finally, the metoestrous phase is defined as a smear with the same proportion of leukocyte, cornified, and nucleated epithelial cells [31].

### 2.6. Histomorphological Analysis of the Ovaries

The ovarian tissues were kept in 10% buffered formalin for 48 h prior to paraffin embedding. Next, paraffin-embedded tissues were sectioned at 5 μm thickness using Leica RM2245 microtome (Leica Biosystems, Wetzlar, Germany), air-dried in a vertical position, dewaxed, rehydrated, and stained with haematoxylin and eosin. The slides were then visualised using an Olympus BX40 light microscope (Olympus Corporation, Tokyo, Japan). The captured image was transferred and analysed with the ImageJ software.

The technique for counting ovarian follicles was adopted from another study [32]. The number of cysts, corpus luteum, and antral and atretic follicles were counted throughout each ovary serial segment. The presence of an oocyte with a nucleus was used to identify healthy follicles. Antral follicles are defined as having two or more layers of cuboidal granulosa cells, whether the cavity is visible or not. The follicles are considered atretic follicles with ovum degeneration or pyknotic granulosa cells. The cystic follicle is a large fluid-filled structure with a thickened theca interna cell layer and an attenuated granulosa cell layer [32].

### 2.7. Statistical Analysis

The data were presented as a mean ± SEM. T-test, one-way ANOVA, or Tukey’s multiple comparison tests were used to determine the differences between the groups using GraphPad Software (GraphPad Inc., San Diego, CA, USA). Statistical significance was defined as *p* < 0.05.

## 3. Results

### 3.1. Effects of Kelulut Honey on the Blood Glucose Levels and Body Weight Gain

**Figure 1:**
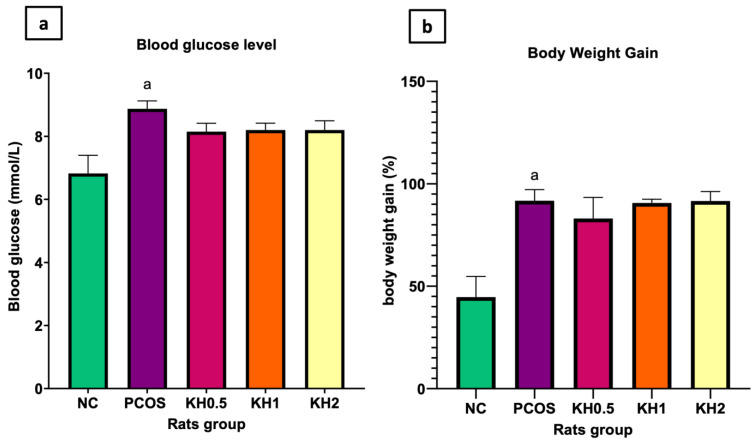
Effects of Kelulut honey on (**a**) blood glucose levels and (**b**) body weight gain. NC: normal control; PCOS: Untreated PCOS; KH0.5: PCOS treated with 0.5 g/kg/day of Kelulut honey; KH1: PCOS treated with 1 g/kg/day of Kelulut honey; KH2: PCOS treated with 2 g/kg/day of Kelulut honey. ^a^ *p* < 0.05 significance against the normal control group. Different colors denote different groups.

Letrozole induction caused the blood glucose levels to be increased compared with normal control (8.87 ± 0.25 vs. 6.825 ± 0.57, *p* < 0.05). Treatment with all three different doses of Kelulut honey did not cause a significant reduction in the blood glucose levels, as shown in Figure 1a. Meanwhile, Figure 1b illustrates the effects of Kelulut honey on body weight gain. The percentage of body weight gain was significantly increased in PCOS rats (91.69 ± 5.48 vs. 44.68 ± 10.05, *p* < 0.05). In contrast, no difference in body weight was observed with the Kelulut honey treatment.

### 3.2. Effects of Kelulut Honey on the Oestrus Cycle

**Figure 2:**
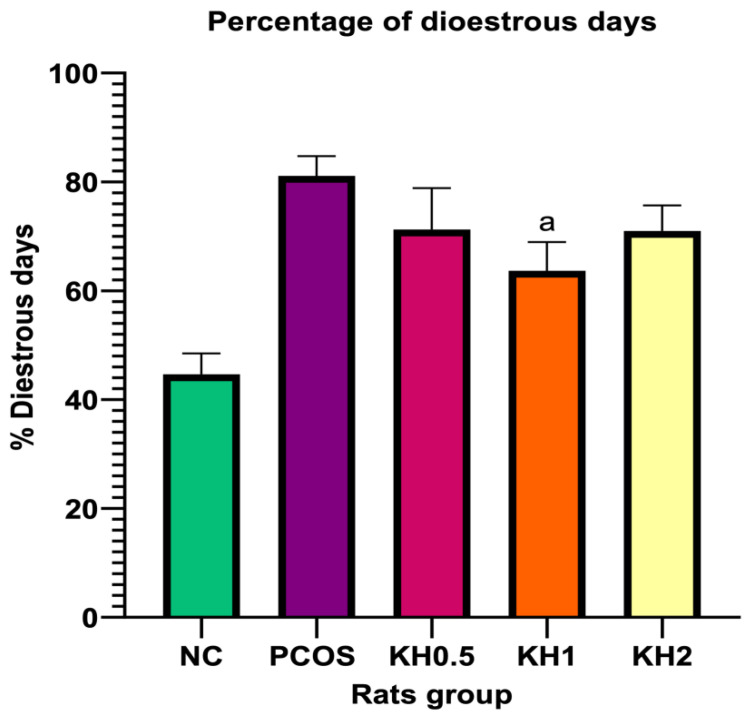
Effects of Kelulut honey treatment on the percentage of dioestrous days. NC: normal control; PCOS: Untreated PCOS; KH0.5: PCOS treated with 0.5 g/kg/day of Kelulut honey; KH1: PCOS treated with 1 g/kg/day of Kelulut honey; KH2: PCOS treated with 2 g/kg/day of Kelulut honey. ^a^ *p* < 0.05 significance against the untreated PCOS group. Different colors denote different groups.

In the present study, rats in the normal control group showed a regular oestrus cyclicity (Figure 2). However, untreated PCOS rats exhibited a significantly higher percentage of dioestrous days than control rats (81.10 ± 3.65 vs. 44.64 ± 3.86, *p* < 0.05). Meanwhile, treatment with 1 g/kg per day of Kelulut honey significantly lowered the percentage of the dioestrous day compared with the PCOS control group (63.68 ± 5.27 vs. 81.10 ± 3.65, *p* < 0.05). In addition, the rat vaginal epithelium staining used to recognise different stages in the oestrus cycle is shown in Appendix B.

### 3.3. Effects of Kelulut Honey on the Ovarian Histomorphological Changes

**Figure 3:**
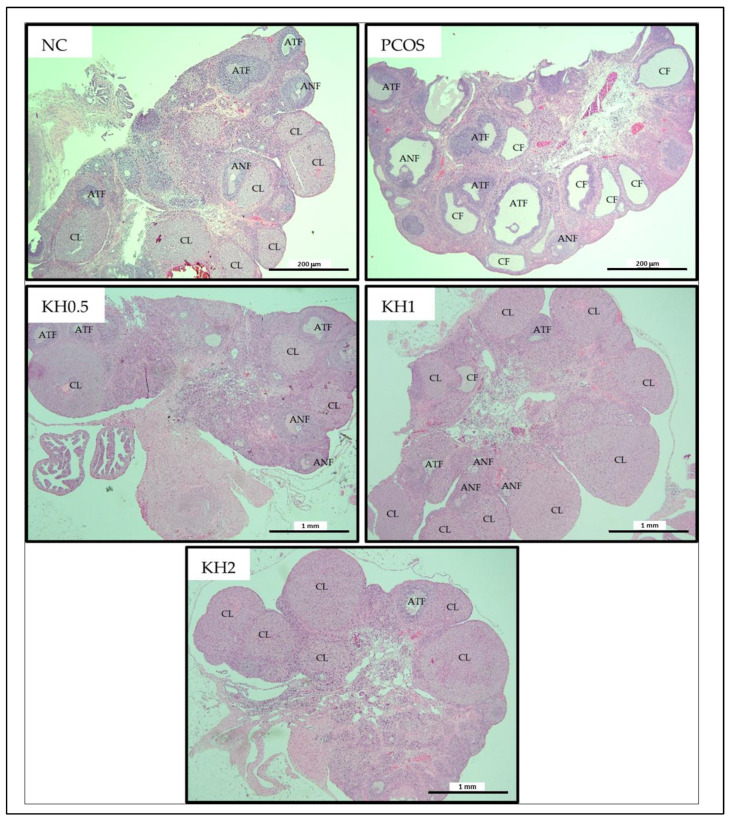
Effects of Kelulut honey treatment on ovarian histology. NC: normal control; PCOS: PCOS Control; KH0.5: 0.5 g/kg/day of Kelulut honey; KH1: 1 g/kg/day of Kelulut honey; KH2: 2 g/kg/day of Kelulut honey; CL: corpus luteum; ANF: antral follicle; ATF: atretic follicle; CF: cystic follicle. Magnification 40×. Scale bar = 200 µm and 1 mm.

Transverse sections of ovaries from the normal control group showed normal histological morphology with appearances of abundant corpus luteum and healthy follicles with a fewer number of atretic and cystic follicles (Figure 3. In contrast, fewer numbers of corpus luteum and antral follicles with numerous follicular ovarian cysts were observed in the untreated PCOS group.

**Figure 4:**
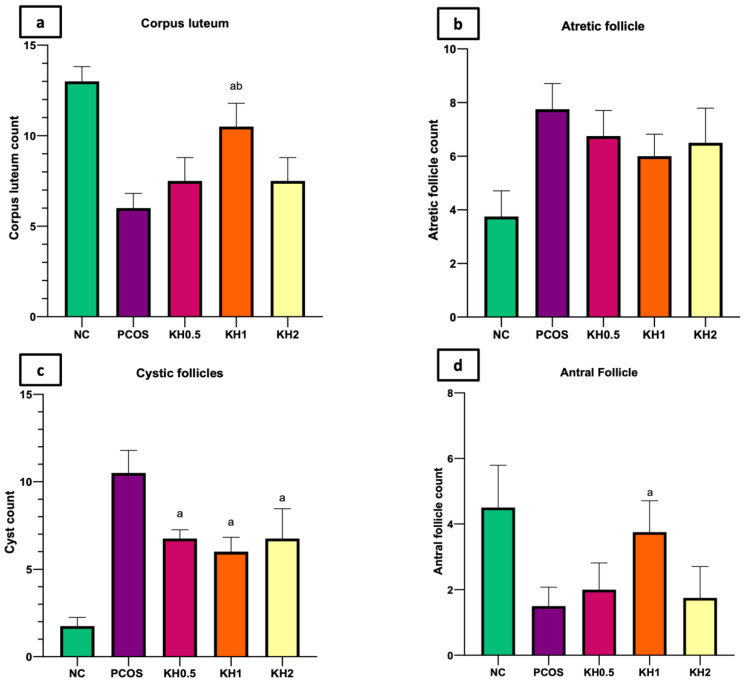
Effects of Kelulut honey treatment on (**a**) corpus luteum (**b**) atretic follicle, (**c**) cystic follicle, and (**d**) antral follicle counts. NC: normal control; PCOS: Untreated PCOS; KH0.5: PCOS treated with 0.5 g/kg/day of Kelulut honey; KH1: PCOS treated with 1 g/kg/day of Kelulut honey; KH2: PCOS treated with 2 g/kg/day of Kelulut honey. ^a^ *p* < 0.05 significance against untreated PCOS group, ^b^ *p* < 0.05 significance against KH0.5 and KH2. Different colors denote different groups.

Treatment with 1 g/kg/day of Kelulut honey significantly increased the corpus luteum (10.5 ± 1.29 vs. 6 ± 0.82, *p* < 0.05) and antral follicle counts (3.75 ± 0.96 vs. 1.5 ± 0.58, *p* < 0.05) while reducing the cystic follicles (6 ± 0.82 vs. 10.5 ± 1.29, *p* < 0.05) compared with the untreated PCOS group (Figure 4). In addition, cystic follicles were reduced in the other two doses of Kelulut honey (0.5 and 2 g/kg/day) compared with the untreated PCOS group (6.75 ± 0.5/6.75 ± 1.7 vs. 10.5 ± 1.29, *p* < 0.05). Additionally, all groups showed an increased (*p* < 0.05) count of atretic follicles compared with the normal control group.

## 4. Discussion

Letrozole, an aromatase inhibitor, effectively induces PCOS in rats [33,34]. It resulted in excess androgens accumulating in the ovary, affecting the follicle development and leading to anovulation and insulin resistance which are the hallmark of PCOS [35,36]. Letrozole-induced PCOS rats also developed hyperandrogenism, ovarian cyst formation, abnormal follicles, hyperglycaemia, and oxidative stress similar to human PCOS [28]. Our study recorded similar findings in PCOS rats, including ovarian cyst formation, atretic follicle, altered oestrus cycle, and hyperglycaemia.

Impaired glucose metabolism is highly prevalent in PCOS women [37,38]. Our current results showed that fasting blood glucose was highest in PCOS rats, which was expected from PCOS induction. Kelulut honey treatment did not reduce the fasting blood glucose in PCOS rats or increase it. A similar finding was reported in a clinical trial, whereby Kelulut honey daily intake for 30 days did not cause any changes in fasting blood glucose in patients with impaired fasting glucose [39].

This study showed that Kelulut honey supplementation did not affect body weight. Honey supplementation has caused a variety of results in body weight. A study originated in Nigeria found that Obudu honey supplementation for 29 weeks causes body weight to increase [40]. A previous study showed that Kelulut honey supplementation decreases the percentage of body weight gain and BMI in a high-fat diet-induced obese rat model more efficiently than orlistat, an anti-obesity drug [41]. Meanwhile, another study found that Kelulut honey might protect rats against metabolic syndrome-induced changes such as increased omental fat mass, triglyceride levels, adipocyte area, and adipocyte perimeter [42]. Obesity has been identified as a risk factor for PCOS [3]. Women with PCOS are overweight or obese in 38–88% of cases [43]. Clinically significant improvements in PCOS characteristics can be achieved with a moderate weight loss of 5–10% [44]. Letrozole treatment significantly increases body weight in treated rats. However, Kelulut honey does not alter the rat weight might be due to each stingless bee honey’s unique botanical origin, determining its nutritional value. In addition, the duration of honey treatment also plays a vital role in inducing body weight alteration [40].

PCOS is mainly associated with ovarian physiological disturbance [45,46]. The letrozole-treated rat’s ovarian histology was comparable to that of human PCOS patients [27]. In both cases, it is feasible to see the follicular arrest, increased number of subcapsular follicle cysts, atretic and decreased thickness of granulosa layer, and absence of corpus luteum [9,27]. In this study, PCOS rats’ ovarian sections exhibited a high number of cystic and atretic follicles and a low count of corpus luteum. These findings were in agreement with several other studies in which PCOS has a detrimental effect on corpus luteum, cystic and atretic follicles [34,47,48]. We showed that 1 g/kg/day of Kelulut honey supplementation significantly increased the corpus luteum count compared to untreated PCOS rats, indicating restoration of ovulation and ovarian function [49].

The presence of cystic follicles was the main finding in PCOS animals [27,28,34]. In this study, cystic follicles were reduced in all Kelulut honey-treated groups. Similarly, in another study, cystic follicles were also decreased with quercetin treatment in PCOS rats [47]. Besides, defective folliculogenesis is one of the PCOS features that is mainly caused by androgen excess [50]. We found that 1 g/kg/day of Kelulut honey treatment significantly reduced antral follicle counts in PCOS rats. This indicates that Kelulut honey brings a closer recovery into the ovary’s physiological state. In another study, antral follicles developed well in PCOS rats upon soy isoflavone treatment [48]. The same trend was recorded in atretic follicles. Although no statistically significant value was recorded, treatment with Kelulut honey reduced the number of the atretic follicle.

The histology of vaginal smears is an important marker of ovarian physiology [51]. In this study, cessation of the oestrus cycle in PCOS rats began on day eight and above, indicating ovarian dysfunction such as cyst formation. Restoration of oestrus cycle was seen in 1 g/kg/day Kelulut honey treatment on PCOS rat, which was in line with its restoration of corpus luteum count. This indicates normalisation toward a physiological state [52]. A similar finding has been reported by Rajan et al. in which the percentage of the diestrous day was reduced with soy isoflavones treatment in PCOS rats [48].

We demonstrated for the first time that Kelulut honey possesses beneficial effects on reproductive disorders in PCOS rats by regaining the oestrus cycle regularities, improving antral follicles and corpus luteum as well, and reducing ovarian cysts. Kelulut honey has been known for its high antioxidative properties [19,20]. Previously, Arentz et al. concluded that various herbal plants might have beneficial effects on PCOS [53,54], which may be due to the antioxidant content possessed by the herbal plants. However, the studies suggest further investigations to support the effectiveness of nutritional supplements and herbal medicine for women with PCOS [53,54]. Kelulut honey’s antioxidative value is contributed by the nectar gathered from various plants collected by the bees [18]. Particularly in this study, Kelulut honey was harvested by a local beekeeper who planted different herbal plants as the source for the stingless bee nectar. Furthermore, honey has benefited female reproductive organs, mainly due to its antioxidant content [17,55]. Hence, the beneficial effect recorded in this study may be attributed to the high antioxidant content of Kelulut honey.

## 5. Conclusions

This preliminary study demonstrates that Kelulut honey, especially at the dose of 1 g/kg/day, can alleviate the reproductive disturbance in PCOS by regaining the oestrus cycle regularities, improving antral follicles and corpus luteum as well, and reducing ovarian cysts. In light of this, Kelulut honey could be further investigated as a complementary treatment for women with PCOS to improve their reproductive disease potentially. However, this present study has its limitations of not determining sex steroid hormone levels, insulin resistance status, and oxidative stress status, as well as elucidating the bioactive compounds of Kelulut honey. Therefore, further studies are needed to explore Kelulut honey’s potential to improve PCOS conditions.

## Figures and Tables

**Figure A3:**
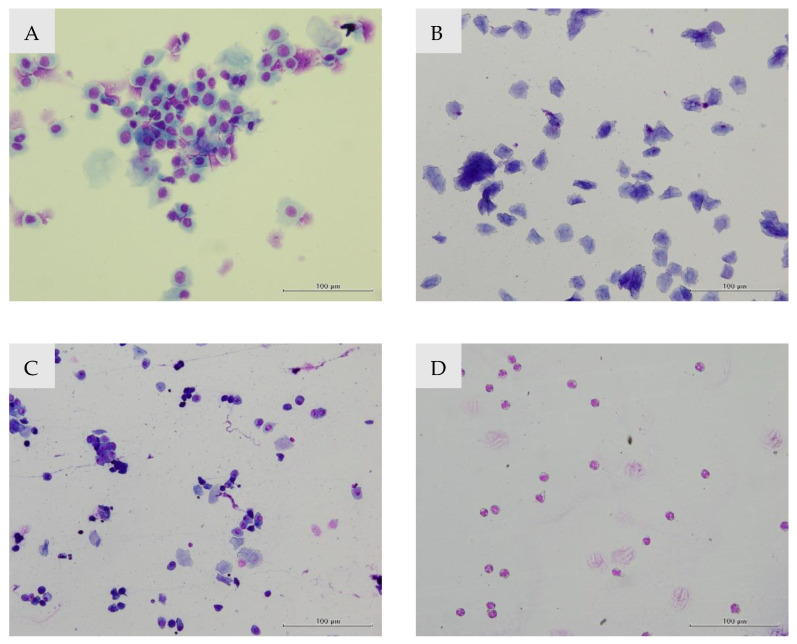
Rat vaginal epithelium staining in different stages of estrus cycle (**A**) proestrus, (**B**) estrus, (**C**) metestrus, and (**D**) diestrus. Magnification 10×. Scale bar = 100 µm.

## Data Availability

The data presented in this study are available within the paper.

## Appendix A

### Effects of Letrozole Treatment on the Percentage of Dioestrous Days, Blood Glucose and Ovarian Histomorphological Parameters

Figure A1 and Figure A2 showed the effects of letrozole treatment on the percentage of dioestrous days, blood glucose levels and ovarian histomorphological parameters. In this study, PCOS was successfully induced in all rats treated with 1 mg/kg/day of letrozole for 21 days. The rats exhibited a prolonged dioestrous stage starting at day eight following letrozole administration. With letrozole treatment, the percentage of dioestrous days was significantly increased compared to the normal control rats (81.10 ± 3.65 vs. 44.64 ± 3.86, *p* < 0.05) (Figure A1a). In addition, letrozole treatment increased the blood glucose levels compared to the normal control group (10.58 ± 0.95 vs. 6.85 ± 0.58, *p* < 0.05) (Figure A1b). Histologically, letrozole treatment significantly reduced the number of corpus luteum (2.0 ± 0.82 vs. 13.0 ± 0.82, *p* < 0.05) (Figure A2a), and antral follicles (0.75 ± 0.5 vs. 4.5 ± 1.29, *p* < 0.05) (Figure A2d), while increasing the number of cystic follicles (13.75 ± 0.96 vs. 1.75 ± 0.5, *p* < 0.05) (Figure A2c) and atretic follicles (9.5 ± 1.29 vs. 3.75 ± 0.96, *p* < 0.05) (Figure A2b).

## Appendix B

### Rat Vaginal Epithelium Staining in Different Stages of Estrus Cycle

Figure A3 showed the rat vaginal epithelium staining in different stages of estrus cycle.
